# Effect of Protein–Oil-Based Emulsion on the Nutritional Value of the Red Deer Meat Sausage

**DOI:** 10.3390/foods15050858

**Published:** 2026-03-04

**Authors:** Eleonora Okuskhanova, Farida Smolnikova, Kumarbek Amirkhanov, Bakhytkul Assenova, Galiya Tumenova, Zhibek Atambayeva, Samat Kassymov, Gulnur Nurymkhan, Assem Spanova, Bakyt Tuganova, Shujaul Mulk Khan

**Affiliations:** 1 Research School of Food Engineering, Shakarim University, 20A Glinki Str., Semey 071412, Kazakhstan; eokuskhanova@gmail.com (E.O.); aspirant57@mail.ru (K.A.); asenova.1958@mail.ru (B.A.); zh.atambayeva@mail.ru (Z.A.); samat-kasymov@mail.ru (S.K.); gulnu-n@mail.ru (G.N.); a.spanova@shakarim.kz (A.S.); 2 Department of Food Security, Kozybayev University, 114 Zhumabayev Str., Petropavlovsk 150000, Kazakhstan; s.tumenov@mail.ru; 3 Department of Biotechnology, Toraigyrov University, 64 Lomova Ave., Pavlodar 140013, Kazakhstan; tuganova65@inbox.ru; 4 Department of Ecology & Plant Sciences, Quaid-i-Azam University, Islamabad 45320, Pakistan; smkhan@gau.edu.pk

**Keywords:** protein–oil emulsion, sausage, red deer (maral) meat, maral fat, fatty acid composition

## Abstract

This study evaluated the feasibility of incorporating a protein–oil emulsion based on beef tripe, meat trimmings, and vegetable oil into semi-smoked sausages produced from maral (red deer) meat, with maral fat used as the sole animal fat source. Four sausage variants were formulated and produced to evaluate the effects of different protein–oil emulsion inclusion levels (0, 10, 15, and 20%) on nutritional, textural, and sensory characteristics. Replacement of part of the maral fat with the protein–oil emulsion resulted in a reduction in total fat content (11.6–14.7%) while protein levels remained stable (20.6–21.4%). Fatty acid analysis demonstrated a significant decrease in saturated fatty acids (from 54.64% in the control to 35.45% in the highest emulsion variant) accompanied by a marked increase in polyunsaturated fatty acids (from 22.20% to 37.57%), primarily due to higher linoleic acid content. Texture profile analysis showed a progressive reduction in hardness, gumminess, and chewiness with increasing emulsion inclusion (*p* < 0.05), whereas springiness and cohesiveness were not significantly affected (*p* > 0.05), indicating the preservation of elastic and cohesive properties. Sensory evaluation confirmed that sausages containing moderate levels of the protein–oil emulsion maintained favorable appearance, flavor, and juiciness, with no adverse effects on overall acceptability. These results indicate that combining maral fat with a protein–oil emulsion is an effective strategy for producing nutritionally improved red deer meat sausages with balanced lipid composition and consumer-acceptable texture and sensory quality.

## 1. Introduction

Traditionally, sausage production has relied on pork bacon fat to provide the fat content, texture, and consistency associated with high-quality products, with optimal fat levels typically reported at 20–35% [1,2]. However, high proportions of pork fat result in elevated saturated fatty acid intake, which has been linked to increased cholesterol levels, obesity, cardiovascular diseases, and other metabolic disorders [3]. In addition, pork-fat-rich sausages are more prone to oxidative rancidity, leading to off-flavors and reduced shelf life during storage [4,5]. These nutritional and technological concerns have driven growing interest in alternative fat sources that can improve lipid quality while preserving sensory attributes and product stability.

In this context, fats derived from wild ungulates, particularly maral (red deer) fat, have attracted attention as a potential alternative to pork fat. Maral fat is characterized by ecological purity and a distinct fatty acid profile shaped by the animal’s natural diet, while retaining the functional properties required for sausage manufacture [6,7]. Nevertheless, like other animal fats, maral fat remains relatively rich in saturated fatty acids and exhibits specific organoleptic characteristics that may limit its use as a sole fat source [8].

Recent research has explored a wide range of strategies for fat substitution. For example, partially replacing pork fat with sunflower oil can yield small-caliber non-acid fermented sausages that exhibit acceptable sensory attributes [9]. Similarly, up to 20% of pork backfat can be replaced with olive oil as a pre-emulsified fat combined with soy protein isolate, maintaining appearance, firmness, odor, and taste comparable to conventional products [10]. In addition, substituting pork backfat with rapeseed or sunflower oil significantly reduces the proportion of saturated fatty acids and increases monounsaturated and polyunsaturated fatty acids in UK-style sausages [11]. Another promising approach involves using collagen and dietary fiber to replace pork back fat in fermented sausages, thereby reducing lipid oxidation and preserving sensory properties during storage [12]. Furthermore, the inclusion of oleogels has been shown to decrease saturated fatty acids, improving both the fatty acid profile and overall sensory properties [13]. Emulsified seed oils have also proved effective, yielding sausages with a softer texture and enhanced fatty acid composition [14]. Moreover, duck fat and β-carrageenan can serve as alternatives to beef fat and pork backfat in frankfurters, improving physicochemical characteristics and oxidative stability without compromising sensory appeal [15].

As the reduction in or elimination of saturated fats becomes increasingly important in meat processing, the selection of suitable fat replacers and functional ingredients is critical. Although pork fat is known for its beneficial content of vitamins A, D, E, and essential micro- and macronutrients, its fatty acid composition is predominantly saturated, posing health challenges when consumed in excess [16]. Diseases of the liver, bile ducts, and gallbladder, as well as cholesterol metabolism disorders, further necessitate lowering saturated fat intake [17]. Thus, identifying and employing innovative fat substitutes holds considerable potential for improving the healthfulness of meat products.

A good option is to use the nutritional and functional benefits of by-products like beef tripe and meat trimmings, along with vegetable oils and milk proteins. Beef tripe is rich in enzymes and micronutrients but contains a high percentage of connective tissue and collagen, influencing its sensory and structural properties [18]. Likewise, meat trimmings share a similar amino acid composition to lean meat but may contain excess connective tissue and lymph nodes, limiting their direct use in premium sausage formulations. With appropriate processing techniques and the formation of protein–oil emulsions, these by-products can help balance vitamin and mineral content, reduce costs, and lower saturated fat content.

Sodium caseinate, derived from casein (the primary milk protein), is widely used in sausage production for its ability to bind fat and water, thereby enhancing product yield and reducing shrinkage. It improves emulsification by stabilizing fat-water mixtures, ensuring consistent texture and minimizing the risk of separation during cooking [19]. In addition, sodium caseinate promotes water-holding capacity, contributing to juiciness and an improved overall mouthfeel in sausages. Its role as a binder also reinforces structural cohesiveness, forming a dense, crosslinked protein network that heightens firmness, chewiness, and sensory quality [20].

Incorporating sunflower oil into sausage formulations is increasingly pursued to improve nutritional profiles. This oil is predominantly composed of polyunsaturated fatty acids (48–74%), mainly linoleic acid (C18:2, omega-6), with moderate monounsaturated fatty acids (14–39%)—primarily oleic acid (C18:1, omega-9), and lower levels of saturated fatty acids (11–13%), such as palmitic (C16:0: 5–7.6%) and stearic acid (C18:0: 2.7–6.5%) [21,22].

By replacing animal fats with sunflower oil, sausages can shift to a higher proportion of unsaturated fatty acids, potentially conferring cardiovascular benefits and aligning with dietary recommendations to reduce saturated fat [23]. Research suggests sunflower oil-based oleogels can enhance cooking yield by retaining moisture, although strategies for structuring oil (e.g., using oleogelators) may be required to address texture and mouthfeel changes [24,25]. Furthermore, unsaturated fats are susceptible to oxidation, underscoring the importance of antioxidants or stabilized oil forms, while formulation and processing adjustments help maintain emulsion stability and water-binding capacity.

The present study focuses on evaluating protein–oil emulsions derived from by-products and vegetable oils as an approach to improving the nutritional, functional, and sensory qualities of red deer meat sausage.

## 2. Materials and Methods

### 2.1. Materials and Reagents

Maral meat (*Cervus elaphus sibiricus Severtzov*) (hip part) was purchased from Bagration farm (Privolnoye village, Ulan district, East Kazakhstan region). Meat trimmings and beef tripe were purchased from meat pavilions in Semey city. Immediately after purchase, the meat and by-products was placed in a portable refrigerated container and transported to the laboratory under controlled temperature conditions of +2 to +4 °C. Sodium caseinate (protein content 90%, ash 4.0%, moisture 6.0%) was purchased from AMK-Chemico (Chelyabinsk, Russia). Vegetable oil (sunflower oil, refined, deodorized) was purchased from food supermarkets in Semey.

### 2.2. Preparation of Protein–Oil Emulsion

The technology of protein–oil emulsion preparation was performed according to the method described in [26]. The emulsion recipe consists of sodium caseinate, finely ground beef tripe and meat trimmings, ultrasonically treated in 2% ascorbic acid solution and vegetable oil (sunflower oil) (Table 1).

Beef tripe and meat trimmings of cattle were pre-cleaned from veins, washed in cold water, cut into small pieces, chopped on a meat grinder (2–3 mm), put into a container and poured into a solution of 2% ascorbic acid to full immersion. The container was placed in an ultrasonic bath PSB-14060-05 (LLC “PSB-Gals”, Moscow, Russia) operating at a frequency of 60 kHz with a generator power of 525 W and a working volume of 14 L, corresponding to a power density of approximately 37.5 W/L. Ultrasonication was applied for 300 s at a controlled temperature of 18–20 °C to minimize thermal effects. The effect of ultrasonic cavitation and oscillating waves of ultrasound leads to the weakening of the structure of collagen fibers of the tripe and trimmings, thereby increasing tenderness. After treatment, it is cooled to a temperature of 2–4 °C and passed through a colloid mill.

Sodium caseinate is added to preheated vegetable oil (25–27 °C) and mixed for 1 min. Further, water is gradually added to the resulting mixture in the process of mixing and continues the process of mixing for 2 min at a frequency of 3000 rpm. At the final stage of mixing finely ground tripe and meat trimmings are added and mixed until a homogeneous viscous consistency is obtained. The obtained additive is used for the preparation of meat products.

### 2.3. Production of Semi-Smoked Sausage from Red Deer Meat

Table 2 presents the formulations of maral meat sausages used in this study, including the control recipe and three experimental variants in which maral fat was partially or completely replaced with a protein–oil emulsion and maral fat. The table summarizes the quantitative composition of the main raw materials and the standardized levels of curing ingredients and spices applied uniformly across all variants.

Semi-smoked sausages were produced using standard meat-processing technology with controlled parameters. Frozen maral meat was received, thawed under refrigerated conditions, and subjected to deboning and trimming to remove connective tissue and visible impurities. The prepared meat was ground using a meat grinder equipped with a 3 mm plate. The minced meat was then salted and cured at 2–4 °C for 12 h.

Following curing, the meat was transferred to a cutter, where maral fat, protein–oil emulsion (according to formulation), and pre-prepared curing ingredients (salt, sodium nitrite, sugar) and spices (black pepper, allspice, garlic) were added and mixed until a homogeneous meat batter was obtained. The resulting batter was stuffed into collagen casings (“Belkozin” LLC, Luga, Russia) with a diameter of 45 mm and formed into sausage links.

Thermal processing was carried out in successive stages: drying at 45 ± 2 °C for 15 min, smoking at 55 °C for 60 min, and cooking at 75 °C for 45 min until the internal temperature at the geometric center of the sausages reached 72 °C. After heat treatment, the sausages were cooled for 2–3 h at a temperature not exceeding 20 °C. The finished products were then subjected to quality control, packaged, labeled, and stored under refrigerated conditions until analysis.

### 2.4. Determination of Proximate Composition

The proximate composition of by-products was determined according to generally accepted methods: total nitrogen content was determined by the Kjeldahl method with the conversion of nitrogen into equivalent protein content using the coefficient 6.25 [27]; moisture content was dried to constant weight at 105 °C [28]; total fat was determined by the Soxhlet method [29]; ash content was determined by dry ashing in a muffle furnace [30].

### 2.5. Determination of Amino Acid Composition

Amino acid composition was determined using a SHIMADZU LC-20 Prominence HPLC system (Kyoto, Japan) with fluorimetric and spectrophotometric detectors. A SUPELCO C18 column (25 cm × 4.6 mm, 5 µm pre-column filter) was used at 40 °C. Eluent gradient mode was employed at 1.2 mL/min. Detection occurred at 246 and 260 nm. Samples underwent acid hydrolysis and derivatization with phenylisothiocyanate (PITC) in isopropanol to form phenylthiohydantoins (PTH). The mobile phase consisted of 6.0 mM CH3SONA (pH 5.5, component A), 1% isopropanol in acetonitrile (component B), and 6.0 mM CH3SONA (pH 4.05, component C). Amino acid standards from Sigma Aldrich (Burghausen, Germany) were used for calibration.

### 2.6. Determination of Fatty Acid Composition

The determination of the fatty acid composition of fat, emulsion and sausages by gas chromatography involves several steps. Samples weighing at least 200 g are ground and stored at 0–5 °C until analysis. Lipids are extracted with a mixture of methanol and chloroform and then concentrated by evaporation. Fatty acids are methylated using acetyl chloride in methanol at 100 °C. The product is neutralized with potassium hydroxide, and the methyl esters are extracted in hexane. The obtained esters are analyzed on a gas chromatograph Crystalux-4000M (Meta-Chrom LLC, Yoshkar-Ola city, Russia) with a capillary column and flame ionization detector, where identification is performed using standard solutions. The results are processed automatically, and the mass fractions of fatty acids are calculated based on the area of their peaks [31].

### 2.7. Determination of Mineral Content

To determine the mineral element content in sausages, approximately 5 g of each sample was weighed using an analytical balance and placed into suitable containers for incineration. The containers were then transferred to a microwave muffle furnace and heated to a final temperature of 600 °C for 12 h to ensure complete ashing of the samples. After cooling, a 10 mL aliquot of a 1:1 (*v*/*v*) hydrochloric acid (HCl) and distilled water solution was added to the ash, and the mixture was stirred thoroughly with a glass rod to dissolve all ash constituents. The resulting solution was filtered through paper filters to remove any remaining particulate matter. The filtrates were subsequently analyzed using an ICP-OES atomic emission spectrometer (Spectro, Boschstr, Burghausen, Germany) to quantify the concentrations of the target mineral elements.

### 2.8. Texture Profile Analysis

Texture Profile Analysis (TPA) was performed using a Brookfield CT3 Texture Analyzer (AMETEK, Middleboro, MA, USA) equipped with a control unit, measuring head, appropriate fixtures, and a portable vertical stage (TA-RT-KIT). Prior to testing, samples were cut into uniform cubes (10 mm × 10 mm × 10 mm), placed in aluminum cylinders, and equilibrated to a temperature of 20–25 °C. The texture analyzer was configured with a compression speed of 5 mm/s and set to compress each sample to 75% of its initial height. Each sample underwent two consecutive compression cycles. From the recorded force-deformation curves, hardness was determined as the maximum peak force during the first compression. Cohesiveness was calculated as the ratio of the area under the second compression curve (A2) to that under the first compression curve (A1), reflecting the sample’s structural integrity after the initial deformation. Springiness, defined as the extent to which the sample recovered its height after deformation, was measured by comparing the distances the probe traveled in each cycle. Adhesiveness was calculated from the negative area of the force-time curve after the first compression, reflecting the work required to overcome the attractive forces between the sample and the probe. Throughout testing, force and deformation data were continuously recorded, ensuring accurate quantification of the textural parameters [32].

### 2.9. Sensory (Organoleptic) Evaluation

Sensory evaluation was conducted in accordance with the requirements of [33,34]. A trained sensory panel consisting of 13 assessors (both genders, aged 25–55 years) participated in the evaluation. All panelists had prior experience in evaluating meat and meat products and were familiar with the sensory attributes of cooked and semi-smoked sausages. Before the study, panelists received refresher training focused on attribute definitions, use of the scoring scale, and evaluation procedures.

The assessment was performed using a 5-point scoring scale, as recommended for sausage products, where 5 = excellent quality and 1 = very poor quality. The following attributes were evaluated: appearance, color on the cut, odor (aroma), consistency, and taste. Samples were coded with random three-digit numerical codes and presented to panelists under blind conditions to avoid identification of formulations. Sausage samples were sliced into uniform pieces and served at 20–22 °C, which corresponds to typical consumption conditions. The serving order of samples was randomized for each panelist to minimize order and carry-over effects. Unsalted crackers and water were provided between samples to neutralize palate perception.

Each panelist evaluated samples independently and recorded scores on standardized evaluation forms. Communication between panelists during evaluation was not permitted. Individual scores were collected and used for subsequent statistical analysis. Mean values and standard deviations were calculated for each sensory attribute.

### 2.10. Statistical Analysis

Statistical analysis was conducted to evaluate differences between the control (sausage with maral fat) and the experimental formulations (sausages containing protein–oil emulsion). For each variant, seven independent sausages (350–450 g) were produced, resulting in a total of 28 sausages. Data were analyzed by one-way analysis of variance (ANOVA) to evaluate the effect of formulation (Variants 1–4). The sausage unit was considered the experimental replicate (*n* = 7 sausages per variant). For each sausage, analytical determinations were performed in triplicate and averaged prior to statistical analysis. When significant differences were detected (*p* < 0.05), mean values were separated using Tukey’s HSD post hoc test. Results are presented as mean ± standard deviation. Statistical analyses were performed using SPSS v. 28, (IBM Corp., Armonk, NY, USA). Differences were considered significant at *p* < 0.05.

## 3. Results

### 3.1. Study of the Proximate and Fatty Acid Composition of Protein–Oil Emulsion

The proximate composition of pork fat, maral fat, and the protein–oil emulsion is presented in Table 3. Both animal fats were characterized by very high fat contents, reaching 92.7% in pork fat and 94.6% in maral fat, with only trace amounts of protein detected in pork fat and no detectable protein in maral fat. Moisture and ash contents in both fats were low and comparable, reflecting their typical role as concentrated lipid ingredients.

In contrast, the protein–oil emulsion exhibited a markedly different composition. Its fat content (37.4%) was significantly lower than that of both pork and maral fats (*p* < 0.05), while protein content increased substantially to 11.8% (*p* < 0.05). This increase is attributed to the inclusion of sodium caseinate and processed animal by-products (beef tripe and connective tissue), which serve as functional protein sources within the emulsion matrix.

The protein–oil emulsion also contained a significantly higher moisture level (50.0%) compared with pork fat (5.4%) and maral fat (4.8%) (*p* < 0.05). In addition, the ash content of the emulsion (0.8%) was slightly but significantly higher than that of both animal fats (0.6%), suggesting a modest increase in mineral content.

Overall, these results demonstrate that the protein–oil emulsion differs fundamentally from both pork and maral fats in its proximate composition, combining reduced fat content with increased protein and moisture. This compositional profile supports its application as a functional fat replacer aimed at improving the nutritional characteristics of maral meat sausages without eliminating the technological functionality required for sausage manufacture.

The fatty acid composition differed markedly among pork fat, maral fat, and the protein–oil emulsion, reflecting distinct lipid sources and nutritional characteristics (Table 4). Pork fat exhibited the highest proportion of saturated fatty acids (SFAs, 36.42%), whereas maral fat showed a slightly lower but still substantial SFA content (34.50%). In contrast, the protein–oil emulsion contained a markedly reduced SFA level (11.82%), indicating a pronounced shift toward a more unsaturated lipid profile. Individual saturated fatty acids such as palmitic (C16:0) and stearic acids (C18:0), which are dominant in animal fats, were substantially lower in the emulsion compared with both pork and maral fats.

Maral fat was characterized by an intermediate fatty acid profile, with balanced contributions of SFAs (34.50%), monounsaturated fatty acids (MUFAs, 25.50%), and polyunsaturated fatty acids (PUFAs, 40.00%). Its PUFA content was notably higher than that of pork fat (23.80%) but lower than that of the emulsion (62.41%), highlighting its potential as a nutritionally improved animal fat source. The elevated PUFA fraction in maral fat was primarily attributable to linoleic acid (C18:2, omega-6), which accounted for 30.81% of total fatty acids, alongside measurable amounts of omega-3 fatty acids, including linolenic acid (C18:3) and docosahexaenoic acid (DHA).

The protein–oil emulsion exhibited the most pronounced enrichment in PUFA, with linoleic acid constituting 62.02% of total fatty acids, more than doubling its proportion in pork fat and substantially exceeding that in maral fat. At the same time, oleic acid (C18:1, omega-9) content was lower in the emulsion (25.26%) than in pork fat (37.93%) but comparable to maral fat (22.50%).

### 3.2. Chemical Composition of Sausages

The chemical composition of semi-smoked maral meat sausages demonstrated formulation-dependent changes associated with the increasing inclusion of the protein–oil emulsion and the corresponding adjustment of maral fat and meat proportions (Table 5). Moisture content increased significantly in Variants 3 and 4 (61.06–61.90%) compared with the control (58.55%) (*p* < 0.05), reflecting the enhanced water-binding capacity of the emulsion system. This increase is technologically relevant, as higher moisture retention may contribute to improved product yield and juiciness without compromising compositional balance.

Protein content remained stable across all formulations (20.60–21.40%), and no statistically significant differences were observed among variants (*p* > 0.05). This indicates that incorporation of the protein–oil emulsion, even at the highest level, did not adversely affect protein density, confirming that the emulsion effectively compensated for changes in fat level while preserving the nutritional protein profile of the sausages.

Fat content decreased significantly with increasing emulsion inclusion. The control sample exhibited the highest fat level (15.60%), while Variants 2, 3, and 4 showed progressively lower values (14.70%, 11.60%, and 13.40%, respectively; *p* < 0.05). The most pronounced reduction was observed in Variant 3, demonstrating that partial replacement of maral fat with the protein–oil emulsion enables a statistically significant reduction in total lipid content while maintaining product structure.

Ash and carbohydrate contents showed no statistically significant differences among formulations (*p* > 0.05). Ash values remained within a narrow range (1.22–1.25%), indicating stable mineral content despite formulation changes. Similarly, carbohydrate content varied only slightly (3.72–3.90%), reflecting minor ingredient redistribution rather than substantive carbohydrate enrichment.

Overall, the results confirm that replacing part of the maral fat with a protein–oil emulsion allows a significant reduction in fat content while maintaining stable protein levels and acceptable moisture balance. Variant 3, in particular, demonstrated the most favorable compositional profile, combining reduced lipid content with increased moisture retention without significant changes in ash or carbohydrate fractions. These findings support the effectiveness of moderate emulsion incorporation as a strategy for producing nutritionally optimized maral meat sausages with preserved compositional stability.

The composition of the emulsion also helped maintain the critical balance of fat required to prevent the dry or rubbery texture that can arise in products with excessive protein and insufficient fat. Research indicates that sausages containing less than 10% fat typically experience notable deficiencies in texture and mouthfeel, which can negatively impact their appeal to consumers [35,36].

### 3.3. Amino Acid Composition of Sausages

When comparing the amino acid profiles of the control (sausage with pork fat) and experimental (sausage with emulsion) samples, several trends emerged in the content of substituted, essential, and total amino acids. Overall, the introduction of the protein–oil emulsion slightly enhanced the total amino acid pool, as well as both substituted and essential amino acids.

The amino acid composition of maral meat sausages demonstrated a clear and formulation-dependent redistribution of both non-essential and essential amino acids following the replacement of pork fat with a protein–oil emulsion. Total amino acids increased from 14,697.8 mg/100 g in the control to 14,876.3–15,058.5 mg/100 g in the modified variants, with the highest level observed in Variant 3, indicating an overall improvement in protein density attributable to the emulsion and collagen-rich components (Table 6).

Among non-essential amino acids, proline and tyrosine showed the most pronounced increases. Proline rose from 762.5 mg/100 g in the control to 859.7 mg/100 g in Variant 3 (*p* < 0.05), reflecting the contribution of connective-tissue proteins and supporting the technological rationale of improving matrix stability. Tyrosine increased by 15.3% in Variant 3 compared with the control (*p* < 0.05), suggesting enhanced incorporation of emulsion-derived proteins. These increments suggest that the substitution of pork fat with the emulsion may contribute to improved structural and functional properties of the protein matrix, as proline and tyrosine are often linked to protein quality and stability [37]. There were also slight increases in serine and glutamic acid, indicating a consistently higher presence of protein-building blocks beneficial to flavor (glutamic acid) and protein hydration (serine) [38]. In contrast, glycine and cystine showed slight reductions, indicating no statistically meaningful loss (*p* > 0.05).

Essential amino acids remained well balanced across all formulations. Phenylalanine and tryptophan increased notably in Variant 3 (*p* < 0.05), while other essential amino acids remained stable, confirming that fat replacement did not compromise protein biological value. These essential amino acids are critical for protein synthesis, muscle maintenance, and metabolic functions [39], suggesting that the emulsion-enhanced sausage could provide a slightly improved essential amino acid profile. Overall, the data demonstrate that replacing pork fat with a protein–oil emulsion not only preserves but modestly enhances the amino acid profile, directly addressing the research objective of developing nutritionally improved maral sausages without diminishing protein quality.

Regarding total amino acids, the content in the experimental samples was higher than in the control sample. This improvement indicates that replacing part of the pork fat with the protein–oil emulsion can slightly enrich the sausage’s overall amino acid density. While all formulations maintained robust amino acid profiles indicative of high-quality protein, the emulsion-substituted product demonstrated incremental increases in both substituted and essential amino acids, as well as a higher total amino acid content. These findings suggest that the protein–oil emulsion approach not only reduces fat content and improves sensory attributes but may also modestly enhance the nutritional value of the finished sausage in terms of its amino acid composition.

### 3.4. Fatty Acid Profile of Sausages

The fatty acid composition of maral meat sausages changed significantly with increasing levels of the protein–oil emulsion and the corresponding reduction in maral fat content (Table 7). The control sample (Variant 1) exhibited the highest proportion of saturated fatty acids (SFAs, 54.64%), reflecting the dominant contribution of maral fat and intrinsic lipids of maral meat. Progressive incorporation of the protein–oil emulsion resulted in a significant and stepwise reduction in total SFA content, decreasing to 47.19% in Variant 2, 40.23% in Variant 3, and 35.45% in Variant 4 (*p* < 0.05). This reduction represents a nutritionally favorable shift and is primarily attributable to the replacement of part of the maral fat with emulsion lipids enriched in unsaturated fatty acids derived from vegetable oil.

At the level of individual saturated fatty acids, palmitic acid (C16:0) and stearic acid (C18:0), which were predominant SFAs in all variants, decreased significantly with increasing emulsion level. Palmitic acid declined from 27.18% in the control to 18.74% in Variant 4 (*p* < 0.05), while stearic acid decreased from 22.64% to 13.24% over the same range (*p* < 0.05). In contrast, long-chain saturated fatty acids (C20:0 and C22:0) increased slightly but significantly with emulsion addition (*p* < 0.05), although their absolute contribution remained low and did not offset the overall reduction in total SFA.

Monounsaturated fatty acids (MUFAs) increased modestly but significantly across formulations, rising from 23.16% in the control to 26.98% in Variant 4 (*p* < 0.05). This increase was mainly driven by oleic acid (C18:1), which showed a consistent and significant rise with increasing emulsion inclusion. The observed trend reflects the combined contribution of emulsion-derived lipids and the redistribution of fatty acids associated with reduced maral fat levels.

Polyunsaturated fatty acids (PUFAs) showed the most pronounced changes among the lipid fractions. Total PUFA content increased significantly from 22.20% in the control to 28.49% in Variant 2, 34.20% in Variant 3, and 37.57% in Variant 4 (*p* < 0.05). This increase was largely driven by linoleic acid (C18:2), whose proportion nearly doubled between the control and Variant 4. In contrast, α-linolenic acid (C18:3) and DHA decreased slightly with increasing emulsion level (*p* < 0.05), reflecting dilution of maral meat–derived omega-3 fatty acids by the vegetable oil component of the emulsion.

Overall, the data demonstrate that incorporation of a protein–oil emulsion in maral meat sausages leads to a statistically significant reduction in saturated fatty acids and a concomitant increase in polyunsaturated fatty acids, particularly linoleic acid, in a dose-dependent manner. Variant 3 represents a balanced formulation, combining a substantial reduction in SFA with a marked increase in PUFA.

### 3.5. Mineral Composition of Sausages

The mineral composition of maral meat sausages showed formulation-dependent changes associated with the replacement of pork fat by a protein–oil emulsion, while maintaining overall mineral balance. Potassium content remained stable in Variants 1–3 (2794.80–2795.89 mg/100 g), indicating that partial fat replacement did not affect potassium retention. In Variant 4, potassium decreased to 2621.54 mg/100 g (*p* < 0.05), likely reflecting the lower proportion of maral meat in this formulation (Table 8).

Calcium content increased from 49.94 mg/100 g in the control to 52.10 mg/100 g in Variant 3 (*p* < 0.05), which can be attributed to the contribution of mineral-rich emulsion components. A relatively modest increase in dietary calcium can contribute to bone health and proper metabolic function, especially when considered as part of a balanced diet [40]. Magnesium showed minimal variation in Variants 1–3 (204.38–204.58 mg/100 g), whereas Variant 4 exhibited a reduction to 192.22 mg/100 g (*p* < 0.05). Magnesium plays an important role in muscle and nerve function, as well as in maintaining a healthy immune system [41]. This minor improvement may be attributed to the animal by-products and vegetable components introduced via the protein–oil emulsion.

Sodium content increased progressively from 561.04 mg/100 g in the control to 577.34 mg/100 g in Variant 3 (*p* < 0.05), reflecting the formulation changes, but decreased in Variant 4. Phosphorus remained stable in Variants 1–3 (703.98–704.97 mg/100 g) and declined significantly in Variant 4 (*p* < 0.05).

Trace elements showed notable enrichment. Iron increased from 0.53 mg/100 g in the control to 0.82–0.91 mg/100 g in Variants 3 and 4 (*p* < 0.05), enhancing nutritional value. Iron is critical for the formation of hemoglobin and the transport of oxygen throughout the body. A higher iron content can be particularly beneficial for individuals who may be at risk of iron deficiency [42]. This increase suggests that the inclusion of beef trimmings and other protein sources in the emulsion may be contributing to enhanced mineral density.

Copper content was not significantly changed in all experimental variants, while zinc remained stable in Variants 1–3 and decreased in Variant 4 (*p* < 0.05). This stability in trace elements indicates that the substitution of pork fat with the protein–oil emulsion had no negative impact on these particular minerals.

In summary, the partial replacement of pork fat with a protein–oil emulsion did not compromise the mineral composition of the sausage and, in fact, led to incremental improvements in calcium, magnesium, and, notably, iron content. These changes align well with the broader goal of creating a more healthful meat product, providing slightly enhanced mineral nutrition while maintaining the overall desired organoleptic and structural properties of the sausage.

### 3.6. Textural Parameters of Sausages

When comparing the textural parameters of the control sausage and the experimental sausages, distinct differences in hardness, gumminess, and chewiness emerge (Table 9). Hardness decreased progressively from the control (58.46 N) to Variant 3 (54.5 N) and Variant 4 (52.5 N), with the control showing significantly higher values than Variants 3 and 4 (*p* < 0.05). Variant 2 (56.0 N) did not differ significantly from the control, indicating that moderate emulsion incorporation did not markedly affect resistance to compression, whereas higher emulsion levels resulted in a softer structure due to partial replacement of the rigid fat phase.

Springiness values ranged narrowly from 0.80 to 0.84 mm, and no statistically significant differences were observed among variants (*p* > 0.05). This indicates that elastic recovery of the sausage matrix was largely preserved across formulations, suggesting that emulsion incorporation did not disrupt the continuity of the protein gel network.

Cohesiveness showed a slight decreasing tendency from the control (0.92) to Variant 4 (0.88); however, differences among formulations were not statistically significant (*p* > 0.05). This trend suggests a modest weakening of internal bonding forces at higher emulsion levels, although overall structural integrity remained acceptable.

Gumminess and chewiness decreased significantly with increasing emulsion content. Gumminess declined from 53.78 N in the control to 46.20 N in Variant 4 (*p* < 0.05), while chewiness decreased from 43.03 to 36.96 N·mm over the same range (*p* < 0.05). These reductions reflect the combined effects of lower hardness and slightly reduced cohesiveness, indicating a softer and less energy-demanding bite at higher emulsion levels.

Overall, the results demonstrate that incorporation of a protein–oil emulsion leads to a controlled softening of the sausage texture without adversely affecting springiness or cohesiveness. Variant 2 maintained textural properties comparable to the control, whereas Variants 3 and 4 exhibited significantly lower hardness, gumminess, and chewiness. These findings indicate that moderate inclusion of the protein–oil emulsion improves the texture of maral meat sausages by increasing tenderness while maintaining elasticity and cohesiveness, which are important for consumer acceptance.

### 3.7. Organoleptic Evaluation of Sausages

**Figure 1:**
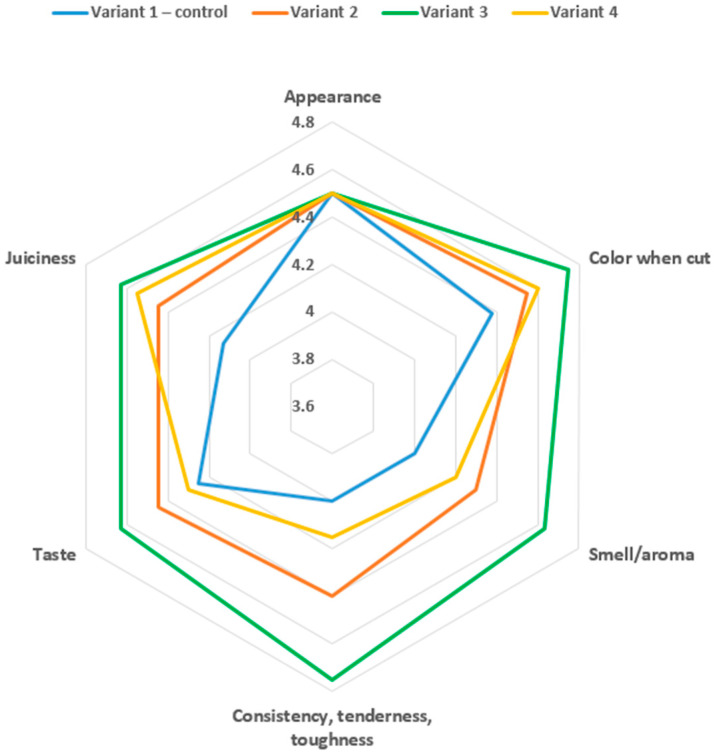
Organoleptic characteristics of sausages.

The organoleptic evaluation demonstrated that partial and complete replacement of pork fat with a protein–oil emulsion and maral fat systematically influenced consumer-relevant sensory attributes. Appearance scores were identical across all variants (4.5), indicating that formulation changes did not affect external visual quality. In contrast, color when cut improved progressively from the control (4.38) to Variant 3 (4.75), representing a significant increase (*p* < 0.05). This enhancement is likely associated with a more homogeneous fat–protein distribution and improved light reflection in the cut surface due to the emulsion (Figure 1).

Smell/aroma scores increased from 4.00 in the control to 4.63 in Variant 3 (*p* < 0.05), suggesting that the replacement of pork fat reduced foreign odor notes and enhanced the characteristic aroma of maral meat. Variant 2 also showed a significant improvement (*p* < 0.05), while Variant 4 exhibited a moderate decrease relative to Variant 3, possibly due to dilution effects at higher emulsion levels.

Consistency, tenderness, and toughness followed trends consistent with instrumental texture data. Variant 3 achieved the highest score (4.75), exceeding the control (*p* < 0.05), reflecting optimal structural integrity and elastic bite. Variant 4 showed a reduced score (4.15), only marginally higher than the control (*p* < 0.05), which may be attributed to lower cohesiveness at the highest emulsion inclusion.

Taste and juiciness were significantly improved in Variants 2 and 3. Taste increased from 4.25 (control) to 4.63 in Variant 3 (*p* < 0.05), while juiciness rose from 4.13 to 4.63 (*p* < 0.05), confirming the positive role of the emulsion in moisture retention and flavor release. Variant 4 retained high juiciness but showed slightly lower taste scores, indicating that excessive emulsion may soften flavor intensity.

**Figure 2:**
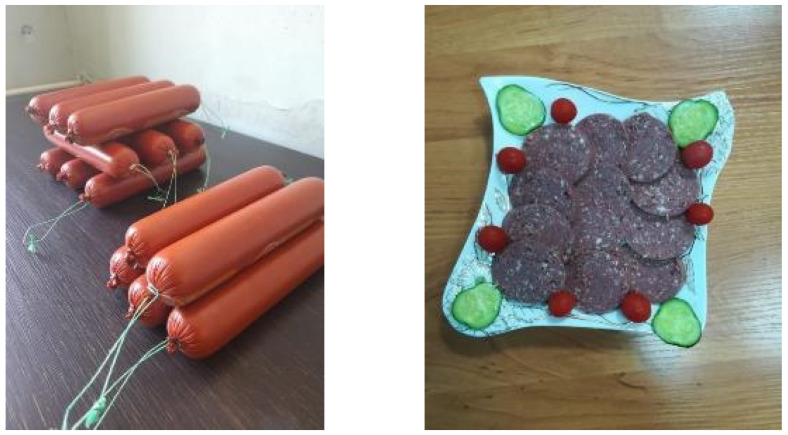
General view and sectional view of red deer meat sausage with protein–oil emulsion.

Overall sensory acceptability increased significantly from 4.21 in the control to 4.65 in Variant 3 (*p* < 0.05), identifying this formulation as the most preferred. Variant 2 also demonstrated a significant improvement in overall rating (*p* < 0.05), while Variant 4, although characterized by high juiciness, showed a slight reduction in preference compared with Variant 3. The sensory panel evaluations indicate that moderate inclusion of the protein–oil emulsion provides a cohesive and desirable texture, improves tenderness, and enhances aroma and taste, despite the absence of conventional pork fat. Collectively, these results confirm that optimized emulsion incorporation (Variant 3) achieves an effective balance of texture, flavor, and juiciness, enabling pork fat replacement without compromising and in some aspects enhancing the overall sensory quality of maral meat sausages (Figure 2).

## 4. Discussion

Emulsions play a key role in meat product processing by stabilizing texture, enhancing nutritional content, reducing cost, and expanding product variety. Replacing animal fats with emulsions can improve structural properties, minimize moisture loss during heat treatment, and preserve palatability [43,44,45]. An adequate proportion of functional proteins is crucial for achieving and maintaining stable emulsions and gels in food systems. Proteins facilitate emulsification by adsorbing at the oil-water interface, thus reducing surface tension and enhancing droplet stability. Simultaneously, they contribute to gel formation by creating a crosslinked network that entraps water and other components. The functional attributes of protein preparations, such as soy isolate or sodium caseinate, influence emulsification, water-binding, and gelling capacities essential to final product quality [46,47].

A comparison of maral meat sausages formulated with a protein–oil emulsion against various studies demonstrates both the versatility and complexity of fat-replacement strategies in meat products. For instance, Bajčić et al. (2023) achieved a drastic reduction in fat content (0.85 mg/100 g) by replacing pork backfat with an inulin-collagen suspension, yet still preserved acceptable texture (hardness 44.15 N, cohesiveness 0.70) [48]. Our sausages (Variant 3), while retaining a higher fat level (11.6%), displayed more pronounced hardness (54.5 N) and cohesiveness (0.90), suggesting that partial fat substitution with a protein–oil emulsion more closely replicates the structural and mouthfeel characteristics of conventional sausages.

In the study replacing 50% of beef fat with safflower oil in wieners [49], reported a relatively high fat content (30.95%) but a lower protein level (13.20%) than ours (21.4%). They observed a significant increase in polyunsaturated fatty acids (PUFAs at 35.29%), albeit accompanied by a much firmer texture [49]. Our formulation (Variant 3) exhibited 34.2% PUFA with a more moderate hardness, reflecting differences in both raw materials and processing conditions. Similarly, the study [50] reported higher fat (17.46%) but lower protein (14.02%) contents in frankfurters produced with pre-emulsified sunflower oil. While the hardness of their product (59.25 N) was comparable to that of Variant 3 in the present study (54.50 N), Clear differences were observed in hardness (59.25 N vs. 54.50 N), cohesiveness (0.68 vs. 0.90) and chewiness (34.08 vs. 39.73 N·mm). These discrepancies are likely related to differences in raw material composition, protein-fat interactions, and moisture distribution between formulations [50].

Further contrasts arise when comparing our results with the study [51], which used an oleogel (sunflower oil structured with beeswax) to replace pork fat, yielding reduced saturated fatty acids (26.37% SFAs) but higher fat (17.90%). Although their approach improved the lipid profile, our emulsion-substituted sausages maintained lower overall fat and higher protein

Meanwhile, substitution of pork backfat with olive oil and rice bran fiber resulted in notably softer meat batters (hardness 3.57 N), highlighting how different protein matrices and fat sources influence textural properties [52]. The work [53] demonstrated that replacing 32.8% of pork fat with a linseed oil gelled emulsion in dry-fermented sausages allowed for higher omega-3 content and moderate protein levels (22.47%), whereas our formulation prioritized omega-6 PUFAs but achieved comparable protein (21.4%). Similarly, the study [54] reported partial replacement of pork backfat with olive and fish oils, resulting in sausages with 17.4% total fat and a lipid profile dominated by monounsaturated fatty acids (MUFAs, 45.59%), while polyunsaturated fatty acids (PUFAs) accounted for 18.58%. In contrast, Variant 3 in the present study exhibited a substantially higher PUFA proportion (34.20%), reflecting the contribution of the protein–oil emulsion. The work [55] reported the potential to entirely replace pork backfat with emulsions high in omega-3 fatty acids while still preserving sensory quality. The study [56] showed that the incorporation of soy oil emulsion gel into deer sausages improves both their nutritional value and flavor, thus presenting healthier alternatives for buyers.

Overall, these varied approaches highlight the trade-offs between drastically lowering total fat versus balancing textural, nutritional, and sensory attributes. Taken together, these findings indicate that partial substitution of maral fat with a protein–oil emulsion in maral meat sausages leads to an improved fatty acid profile, characterized by reduced saturated fat content, while maintaining favorable textural properties.

## 5. Conclusions

This study demonstrated that incorporation of a protein–oil emulsion into maral (red deer) meat sausages is an effective strategy for improving product quality. Partial replacement of maral fat with the emulsion resulted in a favorable modification of the lipid profile, characterized by a significant reduction in saturated fatty acids and an increase in polyunsaturated fatty acids, while protein content remained stable. Textural properties changed with increasing emulsion inclusion: hardness, gumminess, and chewiness decreased, while springiness and cohesiveness were maintained, indicating that the overall structural integrity of the sausages was preserved. Sensory evaluation confirmed that sausages containing moderate levels of the protein–oil emulsion retained desirable appearance, flavor, tenderness, and juiciness, with no negative impact on overall acceptability. Among the tested formulations, Variant 3 (15% protein–oil emulsion) achieved the most balanced combination of nutritional improvement, textural properties, and sensory acceptance, and was identified as the most preferred formulation. Overall, these findings support the potential of combining maral fat with a protein–oil emulsion to produce nutritionally improved red deer meat sausages.

## 6. Patents

Patent #10937 “Method for producing semi-smoked sausage from maral meat using a protein-fat additive” Authors: Okuskhanova E.K., Assenova B.K., Yessimbekov Z.S. Publ. 1 August 2025.

## Figures and Tables

**Table 1:** Formulation of protein–oil emulsion.

Ingredient	Quantity, %
Meat trimmings	10
Beef tripe	10
Sodium caseinate	10
Vegetable oil	35
Water	35
Total	100

**Table 2:** Formulation of maral meat sausages.

Ingredient	Variants, %			
	Variant 1—Control	Variant 2	Variant 3	Variant 4
Maral meat mince	80.0	75.0	75.0	70.0
Protein–oil emulsion	0	10.0	15.0	20.0
Wheat flour	5.0	5.0	5.0	5.0
Maral fat	15	10	5.0	5.0
Total	100.0	100.0	100.0	100.0
Spices, g/100 g				
Salt	2.375	2.375	2.375	2.375
Sodium nitrite	0.006	0.006	0.006	0.006
Granulated sugar	0.1	0.1	0.1	0.1
Ground black pepper	0.075	0.075	0.075	0.075
Ground allspice	0.075	0.075	0.075	0.075
Minced garlic	0.2	0.2	0.2	0.2

**Table 3:** Chemical composition of protein–oil emulsion (mean ± SD).

Indicator	Pork Fat	Maral Fat	Protein–Oil Emulsion
Protein	1.3 ± 0.02	nd	11.8 ± 0.16 *
Fat	92.7 ± 1.39	94.6 ± 1.82	37.4 ± 0.65 *
Ash	0.6 ± 0.01	0.6 ± 0.01	0.8 ± 0.01 *
Moisture	5.4 ± 0.1	4.8 ± 0.1	50 ± 0.9 *

* indicates that values are significantly different within the row (*p* < 0.05). nd—not detected

**Table 4:** Fatty acid composition of protein–oil emulsion (mean ± SD).

Name	Pork Fat	Maral Fat	Emulsion
Sum of saturated fatty acids	36.42	34.50	11.82
Myristic acid C14:0	1.29 ± 0.02 ^b^	2.00 ± 0.02 ^c^	0.26 ± 0.00 ^a^
Pentadecane C15:0	0.07 ± 0.00 ^b^	0.20 ± 0.00 ^c^	0.03 ± 0.00 ^a^
Palmitic acid C16:0	23.24 ± 0.43 ^c^	16.50 ± 0.24 ^b^	5.97 ± 0.07 ^a^
Margarine C17:0	0.38 ± 0.01 ^b^	0.50 ± 0.01 ^c^	0.07 ± 0.00 ^a^
Stearic C18:0	11.26 ± 0.18 ^b^	15.00 ± 0.29 ^c^	4.42 ± 0.05 ^a^
Arachinoic acid C20:0	0.17 ± 0.00 ^a^	0.20 ± 0.00 ^b^	0.31 ± 0.00 ^c^
Behenic C22:0	0.01 ± 0.00 ^a^	0.10 ± 0.00 ^b^	0.76 ± 0.01 ^c^
Sum of monounsaturated fatty acids	39.78	25.50	25.77
Myristoleic acid C14:1	0.01 ± 0.00 ^a^	0.50 ± 0.01 ^c^	0.02 ± 0.00 ^b^
Palmitoleic acid C16:1	1.84 ± 0.02 ^b^	2.50 ± 0.05 ^c^	0.49 ± 0.01 ^a^
Oleic acid C18:1 (omega-9)	37.93 ± 0.67 ^c^	22.50 ± 0.39 ^a^	25.26 ± 0.40 ^b^
Sum of polyunsaturated fatty acids	23.80	40.00	62.41
Linoleic C18:2 (omega-6)	23.04 ± 0.18 ^a^	30.81 ± 0.64 ^b^	62.02 ± 0.61 ^c^
Linolenic C18:3 (omega-6)	0.66 ± 0.01 ^b^	8.72 ± 0.12 ^c^	0.38 ± 0.01 ^a^
Docosahexaenoic acid (DHA) 22:6 (omega-3)	0.10 ± 0.00 ^b^	0.47 ± 0.00 ^c^	0.01 ± 0.00 ^a^
Total	100	100	100

^a–c^ Different lowercase letters indicate statistically significant differences within the rows (*p* < 0.05).

**Table 5:** Chemical composition of sausages (mean ± SD).

Variant *	Moisture, %	Protein, %	Fat, %	Ash, %	Carbohydrate, %
Variant 1—control	58.55 ± 1.13 ^a^	20.70 ± 0.22 ^a^	15.60 ± 0.31 ^d^	1.25 ± 0.03 ^a^	3.90 ± 0.08 ^a^
Variant 2	59.46 ± 1.38 ^ab^	20.80 ± 0.40 ^a^	14.70 ± 0.27 ^c^	1.23 ± 0.02 ^a^	3.81 ± 0.06 ^a^
Variant 3	61.90 ± 0.88 ^b^	21.40 ± 0.31 ^a^	11.60 ± 0.26 ^a^	1.24 ± 0.02 ^a^	3.86 ± 0.04 ^a^
Variant 4	61.06 ± 1.17 ^b^	20.60 ± 0.38 ^a^	13.40 ± 0.22 ^b^	1.22 ± 0.02 ^a^	3.72 ± 0.05 ^a^

^a–d^ Different lowercase letters indicate statistically significant differences within the columns (*p* < 0.05). * Sausage variants differed in the level of protein–oil emulsion inclusion: Variant 1 (control) contained no emulsion, whereas Variants 2, 3, and 4 contained 10%, 15%, and 20% protein–oil emulsion, respectively.

**Table 6:** Amino acid composition of maral meat sausages (mean ± SD).

Amino Acid	Variant 1 (Control) *	Variant 2	Variant 3	Variant 4
Alanine	952.6 ± 7.3 ^a^	943.4 ± 8.4 ^a^	933.2 ± 15.7 ^a^	925.1 ± 15.4 ^a^
Arginine	795.6 ± 12.1 ^a^	798.1 ± 12.6 ^a^	800.0 ± 14.6 ^a^	796.2 ± 14.6 ^a^
Aspartic acid	1051.8 ± 16.6 ^a^	1063.2 ± 13.7 ^a^	1078.2 ± 17.6 ^a^	1069.5 ± 19.9 ^a^
Histidine	721.6 ± 12.0 ^a^	719.2 ± 7.3 ^a^	716.7 ± 17.6 ^a^	712.4 ± 11.6 ^a^
Glycine	706.0 ± 11.2 ^a^	701.6 ± 12.6 ^a^	697.7 ± 12.2 ^a^	692.1 ± 9.9 ^a^
Glutamic acid	1842.4 ± 38.5 ^a^	1853.7 ± 27.1 ^a^	1863.1 ± 22.9 ^a^	1851.9 ± 38.3 ^a^
Proline	762.5 ± 10.9 ^a^	808.4 ± 8.9 ^b^	859.7 ± 8.9 ^c^	835.1 ± 12.2 ^bc^
Serine	494.5 ± 7.1 ^a^	507.2 ± 6.8 ^a^	519.1 ± 5.7 ^b^	512.6 ± 9.6 ^ab^
Tyrosine	549.5 ± 9.5 ^a^	591.2 ± 9.6 ^b^	633.4 ± 9.1 ^c^	610.8 ± 8.6 ^bc^
Cystine	226.1 ± 4.4 ^a^	221.4 ± 4.9 ^a^	215.8 ± 3.8 ^a^	218.7 ± 4.2 ^a^
Hydroxyproline	44.6 ± 0.4 ^a^	43.8 ± 0.8 ^a^	43.1 ± 0.7 ^a^	42.5 ± 0.7 ^a^
Total non-essential AAs	8147.2	8251.2	8360	8266.9
Valine	896.9 ± 17.1 ^a^	907.8 ± 15.7 ^a^	918.8 ± 20.3 ^a^	910.6 ± 13.1 ^a^
Isoleucine	893.8 ± 21.1 ^a^	898.4 ± 16.4 ^a^	902.9 ± 10.5 ^a^	896.3 ± 12.1 ^a^
Leucine	1147.5 ± 21.4 ^a^	1160.2 ± 22.7 ^a^	1172.3 ± 10.9 ^a^	1159.4 ± 15.9 ^a^
Lysine	1486.7 ± 26.0 ^a^	1484.6 ± 21.7 ^a^	1482.9 ± 14.0 ^a^	1471.5 ± 23.4 ^a^
Methionine	510.4 ± 7.3 ^a^	520.9 ± 7.9 ^a^	531.2 ± 11.1 ^a^	523.1 ± 5.3 ^a^
Threonine	821.5 ± 9.9 ^a^	826.3 ± 8.6 ^a^	830.6 ± 12.0 ^a^	823.9 ± 10.5 ^a^
Tryptophan	145.0 ± 2.7 ^a^	149.6 ± 3.8 ^a^	154.0 ± 2.2 ^b^	150.8 ± 2.3 ^ab^
Phenylalanine	648.8 ± 9.7 ^a^	677.3 ± 11.8 ^ab^	705.8 ± 7.3 ^b^	688.4 ± 7.5 ^b^
Total essential AAs	6550.6	6625.1	6698.5	6624
Total amino acids, mg/100 g	14,697.8	14,876.3	15,058.5	14,890.9

^a–c^ Different lowercase letters indicate statistically significant differences within the rows (*p* < 0.05). * Sausage variants differed in the level of protein–oil emulsion inclusion: Variant 1 (control) contained no emulsion, whereas Variants 2, 3, and 4 contained 10%, 15%, and 20% protein–oil emulsion, respectively.

**Table 7:** Fatty acid composition of maral meat sausages (mean ± SD).

Fatty Acid, %	Variant 1 (Control) *	Variant 2	Variant 3	Variant 4
C14:0	2.85 ± 0.05 ^d^	2.34 ± 0.03 ^c^	1.92 ± 0.03 ^b^	1.64 ± 0.03 ^a^
C15:0	0.62 ± 0.01 ^d^	0.51 ± 0.01 ^c^	0.41 ± 0.01 ^b^	0.34 ± 0.01 ^a^
C16:0	27.18 ± 0.42 ^d^	23.95 ± 0.39 ^c^	20.86 ± 0.38 ^b^	18.74 ± 0.28 ^a^
C17:0	0.74 ± 0.01 ^d^	0.61 ± 0.01 ^c^	0.50 ± 0.01 ^b^	0.42 ± 0.01 ^a^
C18:0	22.64 ± 0.47 ^d^	19.02 ± 0.28 ^c^	15.63 ± 0.15 ^b^	13.24 ± 0.29 ^a^
C20:0	0.19 ± 0.00 ^a^	0.21 ± 0.00 ^b^	0.23 ± 0.00 ^c^	0.25 ± 0.01 ^d^
C22:0	0.42 ± 0.01 ^a^	0.55 ± 0.01 ^b^	0.68 ± 0.01 ^c^	0.82 ± 0.01 ^d^
Sum of SFA	54.64	47.19	40.23	35.45
C14:1	0.55 ± 0.01 ^d^	0.46 ± 0.01 ^c^	0.38 ± 0.01 ^b^	0.31 ± 0.01 ^a^
C16:1	2.94 ± 0.05 ^d^	2.45 ± 0.06 ^d^	2.01 ± 0.03 ^d^	1.72 ± 0.01 ^d^
C18:1	19.67 ± 0.46 ^a^	21.41 ± 0.36 ^b^	23.18 ± 0.31 ^c^	24.95 ± 0.42 ^d^
Sum of MUFA	23.16	24.32	25.57	26.98
C18:2	20.03 ± 0.31 ^a^	26.84 ± 0.48 ^b^	32.96 ± 0.49 ^c^	36.58 ± 0.59 ^d^
C18:3	2.00 ± 0.04 ^d^	1.55 ± 0.03 ^c^	1.16 ± 0.02 ^b^	0.93 ± 0.01 ^a^
DHA	0.17 ± 0.00 ^d^	0.10 ± 0.00 ^c^	0.08 ± 0.00 ^b^	0.06 ± 0.00 ^a^
Sum of PUFA	22.2	28.49	34.2	37.57
TOTAL	100	100	100	100

^a–d^ Different lowercase letters indicate statistically significant differences within the rows (*p* < 0.05). * Sausage variants differed in the level of protein–oil emulsion inclusion: Variant 1 (control) contained no emulsion, whereas Variants 2, 3, and 4 contained 10%, 15%, and 20% protein–oil emulsion, respectively.

**Table 8:** Mineral composition of maral meat sausages, mg/100 g (mean ± SD).

Mineral	Variant *			
	Variant 1-Control	Variant 2	Variant 3	Variant 4
Potassium	2795.89 ± 59.64 ^b^	2795.16 ± 43.62 ^b^	2794.80 ± 64.75 ^b^	2621.54 ± 56.10 ^a^
Calcium	49.94 ± 1.25 ^a^	51.38 ± 0.70 ^a^	52.1 ± 1.03 ^a^	50.01 ± 0.73 ^a^
Magnesium	204.38 ± 4.77 ^b^	204.51 ± 2.42 ^b^	204.58 ± 3.32 ^b^	192.22 ± 2.79 ^a^
Sodium	561.04 ± 10.43 ^a^	571.91 ± 6.70 ^ab^	577.34 ± 8.68 ^b^	548.99 ± 7.09 ^a^
Phosphorus	704.97 ± 12.84 ^b^	704.31 ± 10.41 ^b^	703.98 ± 7.65 ^b^	661.52 ± 8.05 ^a^
Iron	0.53 ± 0.01 ^a^	0.72 ± 0.01 ^b^	0.82 ± 0.01 ^c^	0.91 ± 0.01 ^d^
Manganese	0.40 ± 0.01 ^a^	0.41 ± 0.01 ^a^	0.39 ± 0.01 ^a^	0.39 ± 0.01 ^a^
Copper	1.91 ± 0.04 ^a^	1.95 ± 0.03 ^a^	1.97 ± 0.02 ^a^	1.87 ± 0.03 ^a^
Zinc	22.35 ± 0.30 ^b^	22.36 ± 0.41 ^b^	22.37 ± 0.36 ^b^	20.99 ± 0.30 ^a^

^a–d^ Different lowercase letters indicate statistically significant differences within the rows (*p* < 0.05). * Sausage variants differed in the level of protein–oil emulsion inclusion: Variant 1 (control) contained no emulsion, whereas Variants 2, 3, and 4 contained 10%, 15%, and 20% protein–oil emulsion, respectively.

**Table 9:** Texture Profile Analysis (TPA) of sausages (mean ± SD).

Parameter	Variant 1—Control *	Variant 2	Variant 3	Variant 4
Hardness (N)	58.46 ± 0.92 ^b^	56.0 ± 0.92 ^ab^	54.5 ± 0.41 ^a^	52.5 ± 0.95 ^a^
Springiness (mm)	0.80 ± 0.02 ^a^	0.82 ± 0.01 ^a^	0.84 ± 0.01 ^a^	0.81 ± 0.01 ^a^
Cohesiveness	0.92 ± 0.01 ^a^	0.91 ± 0.01 ^ab^	0.90 ± 0.02 ^ab^	0.88 ± 0.02 ^a^
Gumminess (N)	53.78 ± 0.97 ^c^	50.96 ± 0.65 ^b^	49.05 ± 0.45 ^b^	46.20 ± 0.79 ^a^
Chewiness (N·mm)	43.03 ± 0.65 ^c^	41.28 ± 0.44 ^bc^	39.73 ± 0.72 ^b^	36.96 ± 0.67 ^a^

^a–c^ Different lowercase letters indicate statistically significant differences within the rows (*p* < 0.05). * Sausage variants differed in the level of protein–oil emulsion inclusion: Variant 1 (control) contained no emulsion, whereas Variants 2, 3, and 4 contained 10%, 15%, and 20% protein–oil emulsion, respectively.

## Data Availability

The original contributions presented in this study are included in the article. Further inquiries can be directed to the corresponding author.
